# Comparative Performance of Line Probe Assay and GeneXpert in the Detection of Rifampicin Monoresistance in a TB-Endemic African Country

**DOI:** 10.3390/antibiotics11111489

**Published:** 2022-10-27

**Authors:** Betty R. Mchaki, Fauster X. Mgaya, Peter P. Kunambi, Bernard Hang’ombe, Mecky I. Matee, Musso Munyeme

**Affiliations:** 1 Department of Disease Control, School of Veterinary Medicine, University of Zambia, P.O. Box 32379, Lusaka 10101, Zambia; 2 Department of Microbiology and Immunology, Muhimbili University of Health and Allied Sciences, P.O. Box 65001, Dar es Salaam 11103, Tanzania

**Keywords:** Dar es Salaam, diagnostic accuracy, GeneXpert, line probe assay, resistance, rifampicin, Tanzania

## Abstract

Rapid, accurate and reliable assays are required for timely detection of drug-resistant tuberculosis and early initiation of second-line TB treatment as well as to minimize transmission of resistant strains. This study assessed diagnostic performance characteristics of two rapid molecular assays, line probe assay (LPA) and GeneXpert (MTB/RIF), in the detection rifampicin monoresistance using the phenotypic proportion method on Lowenstein–Jensen media as the gold standard. This study involved a total of 357 isolates, 74 rifampicin-resistant and 283 rifampicin-susceptible, collected at the Central Tuberculosis Reference Laboratory (CTRL) in Dar es Salaam, Tanzania, between 2016 and 2019. Sensitivity, specificity and positive and negative predictive values were used to assess the performance characteristics of the two assays while kappa coefficient was used to determine agreement of test results. The receiver operating curve (ROC) was used to determine the discriminatory ability of the test in distinguishing resistant and susceptible TB isolates. Our results showed that GeneXpert had sensitivity, specificity and positive and negative predictive values of 93.2, 82.7, 58.5 and 97.9%, respectively; the corresponding performance for LPA was 86.5, 97.5, 90.1 and 96.5%, respectively. Compared with conventional phenotypic DST results, GeneXpert had a moderate agreement (kappa 0.621, *p* < 0.001), while LPA had high agreement (0.853, *p* < 0.001). LPA showed an accuracy of 95.2% compared to GeneXpert’s 84.9%. ROC curve depicted the ability of the tests to distinguish rifampicin-sensitive and rifampicin-resistant strains to be 87.9% for GeneXpert and 92.0% for LPA. Our results indicate the superiority of LPA over GeneXpert regarding detection of rifampicin monoresistance. However, logistic challenges such as longer turnaround time and need for skilled laboratory personnel may limit its use.

## 1. Introduction

Tanzania is one of the 30 countries with the highest burden of tuberculosis in the world [1]. In 2017, a total of 69,623 cases were reported; 13% of them were children [2]. The prevalence of MDR among new cases ranges from 0.4%, and among recurrent cases it ranges from 3.9% [3]. Meanwhile, the prevalence for any resistance and MDRTB among retreated cases was estimated to be 20.6 and 3.9%, respectively [4,5].

In the year 2020, Tanzania had 1749 TB testing facilities, mostly dependent on smear microscopy, and only 337 GeneXpert machines in the country [6]. The challenges of smear microscopy are well documented and include low sensitivity, especially in all patients with extrapulmonary tuberculosis, resulting in missed cases and delays in initiation of treatment [7]. In Tanzania, drug susceptibility testing is frequently carried out on Lowenstein–Jensen solid media using the proportion method [8,9]. This test method takes weeks before results can be obtained, delaying patient management, hence the need for more rapid and reliable methods with shorter turnaround times.

Currently, the two rapid assays recommended by the WHO are GeneXpert and line probe assay (LPA) [10]. GeneXpert and line probe assay can be used for rapid detection of mutation resulting in rifampicin resistance, while LPA can also detect mutation related to isoniazid resistance [7]. These rapid molecular methods enable early detection and treatment of cases, which is essential in preventing MDR-TB from progression to XDR-TB or, even worse, reaching the TDR-TB level [11]. Comparison of the two techniques in different countries has shown mixed results. For example, some studies show the superiority of GeneXpert and others show that LPA performed better [10,12,13,14].

We conducted our research at the Central Tuberculosis Reference Laboratory (CTRL) in Dar es-Salaam to determine the performance of GeneXpert and LPA in detecting rifampicin resistance using the proportional method on Lowenstein–Jensen (LJ) media as the gold standard. For each method, we calculated sensitivity, specificity and positive and negative predictive values. The kappa coefficient test was used to determine the correlation between the two rapid tests with DST culture results, and the receiver operating characteristic (ROC) curve was used to evaluate the ability of the GeneXpert and LPA methods in distinguishing rifampicin resistance and sensitive isolates.

## 2. Results

Of the 357 isolates tested for rifampicin resistance by the proportional method, 74 were found to be resistant and 283 were found to be sensitive. GeneXpert detected 118 isolates as resistant to rifampicin and 239 as sensitive. Out of 118 rifampicin-resistant *Mycobacteria tuberculosis* (TB) detected by GeneXpert, only 69 were truly resistant. On the other hand, out of 239 rifampicin-susceptible TB isolates detected by GeneXpert, 234 were truly susceptible to rifampicin and the remaining 5 were not susceptible (Table 1).

Out of 71 rifampicin-resistant *Mycobacteria tuberculosis* (TB) detected by the LPA method, only 64 were truly resistant and 7 were false resistant. In addition, out of 284 rifampicin-susceptible TB isolates detected by LPA, 274 were truly susceptible and the remaining 10 were not susceptible (Table 2).

As shown in Table 3, GeneXpert obtained a diagnostic accuracy of 84.9% and a sensitivity and specificity of 93.2% and 82.7%, respectively. The positive and negative predictive values were 58.5% and 97.9%, respectively.

In addition, Table 3 shows that line probe assay had a diagnostic accuracy of 95.2%, with sensitivity and specificity of 86.5% and 97.5%, respectively. The positive and negative predictive values were 90.1% and 96.5%, respectively.

According to kappa testing, the agreement between GeneXpert and DST culture results for the detection of rifampicin was 0.621 (±0.045), *p* < 0.001.

**Figure 1:**
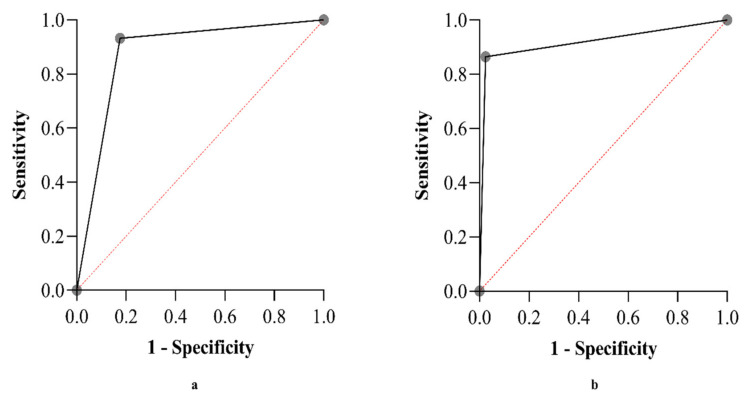
ROC curves depicting performances of GeneXpert (**a**) and LPA (**b**) in detecting rifampicin resistance.

As shown in Figure 1a, which depicts the area under ROC curve, the ability of GeneXpert in detecting rifampicin-resistant TB was 87.9%.

GeneXpert has shown the predictive measures in accuracy of 0.847, specificity of 0.825 and sensitivity of 0.932, with cut-off value set at 0.5.

According to kappa testing, the agreement between LPA and DST culture was high 0.853 (±0.035), *p* < 0.001. The ROC curve shown in Figure 1b indicate that the ability of LPA to detect rifampicin-resistant TB was 92%.

LPA has shown the predictive measures in accuracy of 0.952, specificity of 0.975 and sensitivity of 0.865, with the cut-off value of 0.5.

Table 4 shows significant differences in the results of sensitivities and specificities of GeneXpert and LPA methods in detecting rifampicin resistance using the Miettinen–Nurminen or Tango statistical test.

## 3. Discussion

This study was conducted at CTRL, the national TB reference laboratory for anti-TB drug resistance testing in Tanzania. To the best of our knowledge, there was no previous study conducted in Tanzania that has compared the performance to LPA with GeneXpert in detection of rifampicin resistance. In fact, GeneXpert, which was introduced in the country in 2012, is the only rapid test used in TB testing facilities.

Our study, using the phenotypic proportion method on LJ media as the gold standard in detecting rifampicin resistance, revealed that LPA is better than GeneXpert, as it has a diagnostic accuracy of 95.2%, sensitivity of 86.5%, specificity of 97.5%, positive predictive values of 90.1% and negative predictive values of 96.5%, while GeneXpert has a diagnostic accuracy of 84.9%, sensitivity of 93.2%, specificity of 82.7%, positive predictive values of 58.5% and negative predictive values of 97.9%. The Miettinen–Nurminen statistical test or Tango statistical test that was used to compare sensitivities and specificities of GeneXpert and LPA methods in detecting Rifampicin resistant TB showed statistically significant superiority of the LPA test (Table 4).

Compared with the proportion method [8,9] GeneXpert obtained moderate agreement (kappa 0.621, *p* < 0.001) while LPA obtained high agreement (0.853, *p* < 0.001). Line probe assay showed accuracy of 95.2% compared to GeneXpert’s 84.9%. Our results indicate the superiority of LPA over GeneXpert regarding detection of rifampicin monoresistance.

Our results are comparable with those of a similar study conducted in Kenya that used MGIT as the gold standard, showing GeneXpert’s sensitivity, specificity, PPV and NPV as 62.5%, 96.6%, 62.5% and 96.6%, respectively [9]. In that study, LPA performed better in detecting rifampicin monoresistance, with sensitivity, specificity, PPV and NPV of 90%, 99.1%, 90% and 99.1%, respectively.

Like our study, the Kenyan study found comparatively lower agreement between GeneXpert and culture results (kappa = 0.5905 *p* < 0.001) compared with LPA, which had almost perfect agreement, with sensitivity and specificity of 91.7 and 95.3%, respectively (kappa = 0.8635, *p* < 0.001) [9].

Our results are also in agreement with those of a MGIT 960 study conducted in India [15] that showed 100% agreement with LPA results but only 64.4% agreement with Xpert MTB/RIF results [15]. In that study, subsequent sequencing of discrepant samples showed 91.3% concordance with LPA, but only 8.7% concordance with the Xpert MTB/RIF assay [15].

In operational terms, however, GeneXpert has significant advantages over LPA, having a very short turnaround time (TAT) of 2 to 3 h compared with LPA, which has a TAT of 2 to 3 days. GeneXpert is also easier to perform; unlike LPA, it does not require very skilled laboratory personnel. Thus, detecting rifampicin resistance using LPA may limit testing due to logistic reasons, especially in resource-limited countries, which have the highest burden of TB and TB resistance [16]. To overcome this challenge, we recommend measures to improve the performance of GeneXpert to improve its diagnostic performance in detecting rifampicin resistance. For example, a previous study conducted in Tanzania showed significant improvement of the GeneXpert in diagnosing MTB by lowering the sample reagent to sediment dilution ratio, leading to an increased sensitivity of 6% in MTB detection among presumptive PTB cases, and 12% in HIV-infected individuals [17]. Similar innovative ideas, such as designing country-specific probes, may increase the diagnostic performance of GeneXpert in the detection of rifampicin monoresistance.

## 4. Materials and Methods

### 4.1. Isolates

This study involved a total of 359 isolates collected at the Central Tuberculosis Reference Laboratory (CTRL), Tanzania, between 2016 and 2019.

### 4.2. Drug Susceptibility Testing on Lowenstein–Jensen Media

This was performed using proportional method [8] on Lowenstein–Jensen (LJ) media containing 0.2 µg/mL and 1.0 µg/mL of isoniazid, 5 µg/mL streptomycin, 40 µg/mL of rifampicin, 2 µg/mL ethambutol and 500 ug/ml para-nitrobenzoic acid (PNB) [18]. The proportion method determines the percentage of growth (number of colonies) of defined inoculums on a drug-free control medium versus growth on culture media containing the critical concentration of an anti-TB drug. The proportion method enables precise quantification of the proportion of organisms resistant to a given drug. First reading is done at 4 weeks (28 days). If resistant, no further reading, while if no growth seen, re-incubation was done up to 42 days [19].

### 4.3. GeneXpert

We used isolates for processing in GeneXpert; hence, there was no need for decontamination process. The buffer used was sample reagent (SR) buffer (Pro-Gen Diagnostics) by mixing 1 mL of isolates in sterile distilled water with 2 mL of sample reagent. Between 2–3 colonies of TB isolates were added to the falcon tube with 1 mL of sterile distilled water, and then 2 mL of sample reagent was added and shaken vigorously 10–15 times, followed by incubation at room temperature for 10 min. The shaking process was repeated, samples were incubated for another 5 min, and then the samples were ready to be loaded in the GeneXpert MTB/RIF cartridges as per manufacturer’s instruction [20,21]. Using a sterile pipette, 2 mL of liquefied sample was added in the cartridges, which were now ready to be loaded in the GeneXpert instrument. This was carried out according to the manufacturer’s instructions [22,23,24,25].

### 4.4. Line Probe Assay (LPA)

The procedures for DNA extraction, amplification, hybridization and interpretation were performed according to the manufacturer’s instructions [26], as outlined below.

### 4.5. DNA Extraction

A total of 300 uL of sterile molecular grade water was pipetted into 1.5 mL vial, using 1 uL sterile inoculation loop; 1–2 bacterial colonies were scooped and put in that 1.5 mL vial. Then, DNA extraction was performed on the isolates using GenoLyse extraction procedure [27].

### 4.6. Amplification

Master mix was prepared by adding 10 uL of Amplification mix A (AM-A), then adding 35 uL of Amplification B (AM-B), and then 5 uL of DNA template was added [28]. The profile for amplification in the thermo cycler was denaturation at 95 °C for 15 min for 1 cycle, followed by annealing at 65 °C for 2 min for 10 cycles, then extension at 70 °C for 40 s for 20 cycles and, finally, elongation at 70 °C for 8 min for 1 cycle.

### 4.7. Hybridization and Detection

After denaturation of the amplicons, 1 mL of the pre-warmed hybridization buffer (HYB) was carefully added to the wells using a pipette and thoroughly mixed. The tray was placed on the TwinCubator^®^ and labelled strips were added to each well, ensuring that the strips were completely covered by the liquid, and incubated at 45 °C for 20 min. After incubation, the HYB buffer was aspirated completely from each well and 1 mL of the pre-warmed red stringent wash buffer (STR) was then dispensed into the tray. After 10 min incubation at 45 °C in the TwinCubator^®^, STR buffer was aspirated and was washed off with 1 mL of rinse solution (RIN) for 1 min. Then, 1 mL of the conjugate (CON) solution was dispensed into each well and incubated for 20 min on the TwinCubator^®^. The strips were washed twice with 1 mL of rinse solution (RIN) for 1 min in the TwinCubator^®^. Then, sterile distilled water was added and a 1 min wash performed on the TwinCubator^®^ to wash off the RIN solution, after which the distilled water was completely decanted. An amount of 1 mL of the substrate solution was then dispensed into each well and incubated for 10 min on the TwinCubator^®^; subsequently, the substrate solution was aspirated and the strips washed twice with sterile distilled water. A pair of clean tweezers was used to remove the strips from the TwinCubator^®^ tray and place them onto absorbent paper. The developed strips were partially dried and transferred to the GenoType^®^ MTBDRplus score sheet for interpretation as either sensitive, resistant or invalid [25,29,30].

### 4.8. Statistical Analysis

Data were analyzed using R (Version 4.2.0) with its integrated development environment (IDE), referred to as JAMOVI version 2.2, together with Excel extensions “Analyse it version 6.0” [28]. Sensitivity, specificity and positive and negative predictive values of GeneXpert and LPA in detecting rifampicin-resistant TB were determined using DST culture results as the gold standard. To compare sensitivities and specificities of GeneXpert and LPA methods in detecting rifampicin-resistant TB, the Miettinen–Nurminen statistical test or Tango statistical test was used. The test provided score confidence interval, and a Score Z test, which was used under hypothesis of equality, showed that “the sensitivities/specificities of two tests are equal”. Kappa statistics were used to assess degree of agreement between GeneXpert and LPA with DST culture in detecting rifampicin-resistant TB. The receiver operating curve (ROC) was used to determine discriminatory ability of tests in distinguishing resistant and susceptible TB isolate [28,31].

## 5. Conclusions

Results of this study showed the clear superiority of LPA over GeneXpert in the detection of rifampicin monoresistance. All parameters used to evaluate diagnostic accuracy (sensitivity, specificity, PPV, NPV and ROC) favored LPA. Compared with the proportion method, GeneXpert had moderate agreement (kappa 0.621, *p* < 0.001) while LPA had high agreement (0.853, *p* < 0.001). Line probe assay showed a diagnostic accuracy of 95.2% compared to GeneXpert’s 84.9%. However, logistical issues such as longer turnaround time and requirement of trained laboratory personnel may limit the use of LPA, especially in low-income countries, where the burden of TB- and rifampicin-resistant TB is highest in the world.

## Figures and Tables

**Table 1:** Comparison of rifampicin resistance results of conventional culture method versus GeneXpert.

	Culture-Drug Susceptibility Test		
GeneXpert	Resistance	Susceptible	Total
Resistance	69	49	118
Susceptible	5	234	239
Total	74	283	357

**Table 2:** Comparison of rifampicin resistance results of conventional culture method versus line probe assay.

	Culture-Drug Susceptibility Test		
Line Probe Assay	Resistance	Susceptible	Total
Resistance	64	7	71
Susceptible	10	274	284
Total	74	281	355

**Table 3:** Diagnostic performance of GeneXpert and LPA in diagnosing rifampicin-resistant *Mycobacterium tuberculosis* isolates.

	Gene Xpert		LPA	
Decision Statistics	Estimate	95% CI	Estimate	95% CI
Test sensitivity	93.2%	84.9–97.8%	86.5%	76.5–93.3%
Test specificity	82.7%	77.8–86.9%	97.5%	94.9–99.0%
Diagnostic accuracy	84.9%	80.7–88.4%	95.2%	92.4–97.2%
Positive predictive value	58.5%	49.0–67.5%	90.1%	80.7–95.9%
Negative predictive value	97.9%	95.2–99.3%	96.5%	93.6–98.3%

**Table 4:** Comparison of sensitivities and specificities of GeneXpert and LPA methods in detecting rifampicin resistance.

	Proportion Difference	Tango 95% CI	Z Statistic	*p*-Value
Sensitivity	−0.068	−0.149 to −0.015	−2.24	0.0253 ^$^
Specificity	0.151	0.114 to 0.198	6.48	<0.0001 ^$^

^$^ Reject the null hypothesis in favor of the alternative hypothesis (inequality) at the 5% (0.05) significance level.

## Data Availability

The data presented in this study are available upon reasonable request from the corresponding author.
